# Epigenetic Regulation in Gastroenteropancreatic Neuroendocrine Tumors

**DOI:** 10.3389/fonc.2022.901435

**Published:** 2022-06-07

**Authors:** Judy S. Crabtree

**Affiliations:** Department of Genetics, Louisiana State University Health Sciences Center, New Orleans, LA, United States

**Keywords:** DNA methylation, neuroendocrine, histone modification, miRNA, pancreatic

## Abstract

Gastroenteropancreatic neuroendocrine neoplasms are a rare, diverse group of neuroendocrine tumors that form in the pancreatic and gastrointestinal tract, and often present with side effects due to hormone hypersecretion. The pathogenesis of these tumors is known to be linked to several genetic disorders, but sporadic tumors occur due to dysregulation of additional genes that regulate proliferation and metastasis, but also the epigenome. Epigenetic regulation in these tumors includes DNA methylation, chromatin remodeling and regulation by noncoding RNAs. Several large studies demonstrate the identification of epigenetic signatures that may serve as biomarkers, and others identify innovative, epigenetics-based targets that utilize both pharmacological and theranostic approaches towards the development of new treatment approaches.

## 1 Introduction

**Figure 1:**
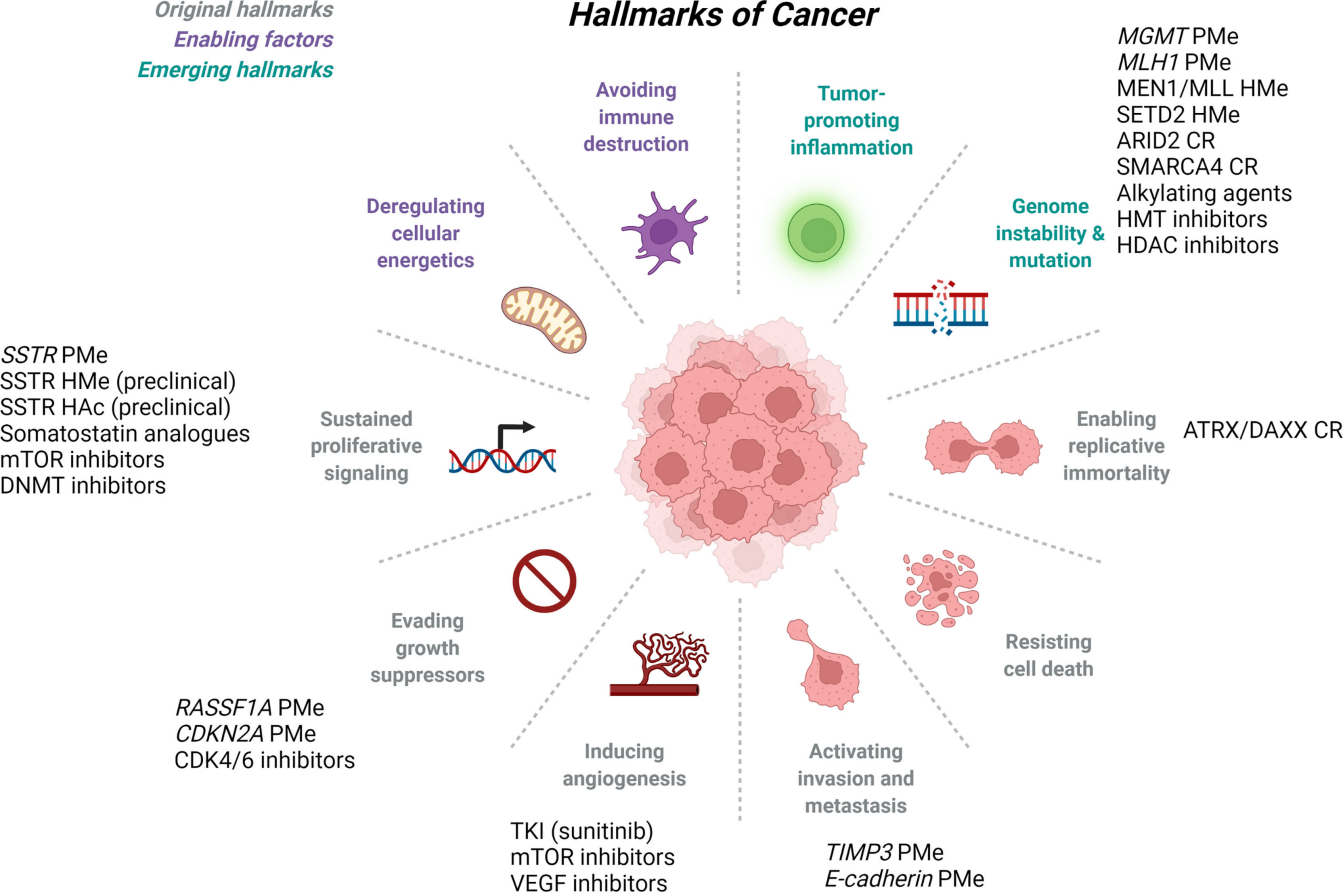
*Hallmarks of Cancer and Epigenetic Regulation of GEP-NETs*. The original Hallmarks of Cancer as published in 2000 by Hanahan and Weinberg (gray), were updated in 2011 to include additional enabling factors (purple) and emerging hallmarks of cancer (teal). GEP-NETs have been studied in several of the defined hallmarks of cancer, and listed here are the epigenetic mechanisms and therapeutics associated with each. ARID2, AT-rich interactive domain 2; ATRX, alpha-thalassemia/mental retardation, X-linked; CDK4/6, cyclin dependent kinase 4/6; CDKN2A, cyclin dependent kinase inhibitor 2A gene; CR, chromatin remodeling; DAXX, death domain-associated protein; DNMT, DNA methyltransferase; HAc, histone acetylation; HDAC, histone deacetylase; HMe, histone methyltation; HMT, histone methyltransferase; MEN1, multiple endocrine neoplasia type 1; MGMT, O-6-methylguanine DNA methyltransferase gene; MLH1, mutL homolog 1; MLL, mixed lineage leukemia lysine methyltransferase 2A; mTOR, mammalian target of rapamycin; PMe, promoter methylation; RASSF1A, ras associated domain family 1 gene; SETD2, set domain-containing protein 2; SMARCA4, SWI/SNF-related matrix associated actin dependent regulator of chromatin subfamily a, member 4; SSTR, somatostatin receptor; TIMP3, tissue inhibitor of metalloproteinase 3 gene; TKI, tyrosine kinase inhibitor; VEGF, vascular epithelial growth factor. Adapted from “Hallmarks of Cancer” by BioRender.com (2022), Retrieved from https://app.biorender.com/biorender-templates.

Gastroenteropancreatic neuroendocrine neoplasms (GEP-NENs) have steadily increased in prevalence and incidence, and this number is expected to continue rising primarily due to improvements in diagnostic imaging and physician awareness (1–3). Patients with GEP-NENs often present with advanced disease at diagnosis and surgery alone is rarely curative (4). Despite association of GEP-NENs with several genetically linked syndromes, the molecular mechanisms of GEP-NENs are not well understood. Recent studies have provided evidence for the importance of particular cellular processes such as angiogenesis, or pathways such as G-protein coupled receptor (GPCR) activation in the pathogenesis of GEP-NENs, but there remains significant work to be done. Hanahan and Weinberg outlined the “Hallmarks of Cancer” first in 2000 (5), then again in 2011 (6) that included the cancer-driving categories of tumor-promoting inflammation, genome instability and mutation, enabling replicative immortality, resisting cell death, activating invasion and metastasis, inducing angiogenesis, evading growth suppressors, sustained proliferative signaling, deregulating cellular energetics, and avoiding immune destruction. Studies on GEP-NENs suggest that the generalized Hallmarks of Cancer also apply to this tumor type [**Figure 1** and reviewed in (7)] and many of these categories will be discussed below as they related to epigenetic regulation of GEP-NENs. The goal of this review is to briefly touch on the current therapeutic options for patients with GEP-NENs [these have been extensively reviewed in (8–11)], then explore details of the emerging epigenetic approaches in terms of utility as diagnostic or prognostic biomarkers, or therapeutics for this rare tumor type.

## 2 Current Clinical Approaches

Neuroendocrine neoplasms (NENs) in general are clinically and biologically diverse tumors that arise in a variety of tissues including pituitary, parathyroid, lung, skin, pancreas and gastrointestinal tract. The diversity of these tumors presents challenges when establishing tumor classification categories and patient management guidelines, and the nomenclature has changed several times over the last several decades. Recently, the guidance for these tumors has begun to stabilize as a result of significant efforts by the World Health Organization and other regulatory bodies, allowing pathologists and clinicians a more consistent taxonomy. Currently, NENs are divided into three major classes including 1) neuroendocrine tumors (NET) which included well differentiated tumors, 2) neuroendocrine carcinoma (NEC) which are poorly differentiated tumors, and 3) mixed adenocarcinoma (MANECs or MiNEN; mixed neuroendocrine-non-neuroendocrine neoplasm) (12).

A subset of NENs are the gastroenteropancreatic neoplasms (GEP-NENs) that are within themselves a very heterogeneous group of tumors arising from the neuroendocrine cells of the pancreas and gastrointestinal tract. Well differentiated GEP-NETs are classified as either Grade 1 if they demonstrate <3% Ki67 index and mitoses <2 per 10 high-power visual field, or Grade 2 if they have 3-20% Ki67 index and mitoses 2-20 per 10 high-power visual field. All poorly differentiated GEP-NECs are classified as Grade 3, meaning >20% Ki67 index and >20 per 10 high-power visual field. MANECs are defined as having >30% of each component in the tumor and were defined as NEC Grade 3 containing non-neuroendocrine components (typically adenocarcinoma) (13).

In terms of treatment, surgical resection is often the first line therapy for either curative (for localized, non-metastastic cancer) or palliative (for advanced metastatic disease) care, followed by pathway-specific chemotherapeutic approaches that are administered systemically. For patients with advanced disease and functional tumors, controlling symptoms as a result of hormone hypersecretion becomes paramount. Somatostatin analogs (SSA) such as octreotide, lanreotide and the second generation compound pasireotide are typically the first line therapy for symptom control. These drugs work by binding tightly to one of several somatostatin receptors (SSTR) which have variable expression both across different tumor types and also within the same tumor type. In general, 70-90% of GEP-NENs express SSTR2, followed by SSTR5 (14, 15). The efficacy of SSA treatment is dictated by the amount and subtype of SSTR expressed by the tumor. SSAs have been rigorously tested in clinical trials, including the PROMID (16) and CLARINET (17) trials, which demonstrated improved symptom control, tumor growth control and progression free survival (PFS) compared to placebo.

Peptide receptor radionuclide therapy (PRRT) is the systemic delivery of cytotoxic radionuclides that specifically target cells expressing high levels of SSTR. The most commonly used approach is (177)Lu-DOTATATE that was studied in the NETTER-1, phase III trial (18, 19). In this trial of midgut neuroendocrine tumors, PRRT treatment provided superior PFS, and improved response rates, symptom control and quality of life. Additional trials that included pancreatic NETs, also demonstrated increased PFS and overall survival (OS), along with improved symptom control and quality of life (20, 21).

The mammalian target of rapamycin (mTOR) pathway has also been a target of systemic therapies for GEP-NENs. The mTOR inhibitor everolimus was used in the RADIANT-3 (22) and RADIANT-4 (23) trials and demonstrated increased PFS compared to placebo, and similarly, sunitinib, a broad spectrum tyrosine kinase inhibitor showed superiority in PFS compared to placebo in a phase III trial (24). There is no consensus on the use of cytotoxic chemotherapy such as 5-fluorouracil, platinum drugs, or alkylating agents, but some combinations have yielded durable response rates in GEP-NENs, and additional trials such as the SENECA trial (NCT03387592) are ongoing to address these systemic therapies. Comprehensive reviews on drug therapy for GEP-NENs have been published recently (8–11).

## 3 Genetic Basis of GEP-NENs

GEP-NENs most often arise as sporadic tumors, but roughly 10% of pancreatic NETs (pNETs) occur in hereditary syndromes like multiple endocrine neoplasia, type I (MEN1) (25), Von Hippel-Lindau (VHL) (26), neurofibromatosis type I (NF1) (27) and tuberous sclerosis complex (TSC) (28). MEN1 is associated with two or more tumors of the parathyroid, pituitary, and GEP-NENs and is associated with germline mutations in the *MEN1* gene on chromosome 11q13 (25). While more than 1300 germline mutations have been identified to date, sporadic mutations can also occur. MEN1 has an important role in cellular proliferation pathways and is reported to impact Wnt/β-catenin (29) and hedgehog (30) signaling, NF-κB transactivation (31), MAPK-ERK signaling (32) and PI3K-mTOR-Akt signaling (33). VHL is another tumor syndrome that phenotypically includes pNETs along with pheochromocytoma, renal cell carcinoma and hemangioblastoma and results from mutation in the *VHL* gene on chromosome 3p25 (26). It is thought that mutated VHL facilitates degradation of hypoxia inducible factor 1, which induces production of growth factors, angiogenesis and tumor growth (34). TSC arises from mutations in the *TSC1* gene on chromosome 9q34 or *TSC2* gene on chromosome 16p13.3 that lead to increased proliferation by activation of the mTOR signaling pathway and includes phenotypic skin abnormalities, renal system angiomyolipomas, hamartomas and neurological defects. Pancreatic neuroendocrine carcinoma are a rare part of this genetic syndrome (35). Finally, NF1 arises from mutations in the *NF1* gene located on chromosome17q11.2 that result in activation of the RAS-MAPK and PI3K-mTOR pathways. Patients with these mutations develop a spectrum of cancers including myeloid leukemia, pheochromocytoma, rhabdomyosarcoma, central nervous system tumors and GEP-NENs, including pNETs (36).

Recent large scale studies have been performed to define pNET genetic abnormalities (37–40). Using whole exome sequencing of 10 nonfunctional, sporadic pNETs followed by directed sequencing of an additional 58 pNETs, Jiao, et al. confirmed the frequency of *MEN1* mutations in 40% of cases, inactivation of *TSC1/2* in 6% of cases and dysregulation of the P13K/mTOR signaling pathway by identifying *PTEN* inactivating mutations in 5% of cases. Scarpa, et al. performed a study on 102 primary pNETs and reported new germline mutations in DNA repair genes *MUTYH*, *CHEK2* and *BRCA2*, along with additional somatic mutations involved in chromatin remodeling, mTOR signaling (new *EWSR1* gene fusions), and hypoxia (38).

A study completed in 2022 by Simon et al. performed in-depth genomic analysis of 57 pNENs (43 pNET, 14 pNEC) using DNA sequencing to identify recurrent genetic mutations. From this analysis, the authors identified two groups: Group A included mutations that are often associated with pNETs including *MEN1*, *DAXX*, *ATRX*, *VHL*, *PTEN* and *TSC2*, while Group B contained no mutations in *DAXX*, *ATRX* or *MEN1* but contained *KRAS*, *SMAD4* and *TP53* mutations (39). Allen, et al. investigated the clinical significance of *BRAF* alterations in a series of 80 well-differentiated, metastatic pNETs. *BRAF* mutations were identified in 6 samples (7.5%): two harbored V600E, one tumor each for K601E, T599K and T310I mutations, and the final tumor carried both G596D and E451K mutations (41). *In vitro* studies suggested that these mutations may serve as biomarkers for therapy response to RAF and MEK inhibition in this small subset of pNET tumors (41). NTRK fusions have also been investigated in NENs and in a study of 2417 tumors, NTRK alterations were identified in six cases (0.3%; 2 pancreas, one uterus, one lung and 2 unknown origin) (42). Fusion partners included an intergenic region, *PIP5K1A*, *CCDC19*, *GPATCH4*, *ETV6* and *SQSTM1*.

Gastrointestinal neuroendocrine neoplasms (GI-NENs) have also been analyzed using large scale DNA sequencing to uncover genomic and epigenomic alterations. Yachida, et al. studied 115 cases of GI-NENs using whole genome/exome sequencing. The collection of 60 GI-NECs analyzed included pancreatic, colorectal, gastric, biliary, ampullary and esophageal tumors, while the remaining 55 GI-NET samples included pancreatic, colorectal and duodenal. Analysis of mutations allowed GI-NECs to be distinguished from GI-NETs, with GI-NECs containing mutations in *TP53*, *KRAS*, *RB1*, *CCNE1*, *CDKN2A* and *MYC (* 43).

## 4 DNA Methylation

**Figure 2:**
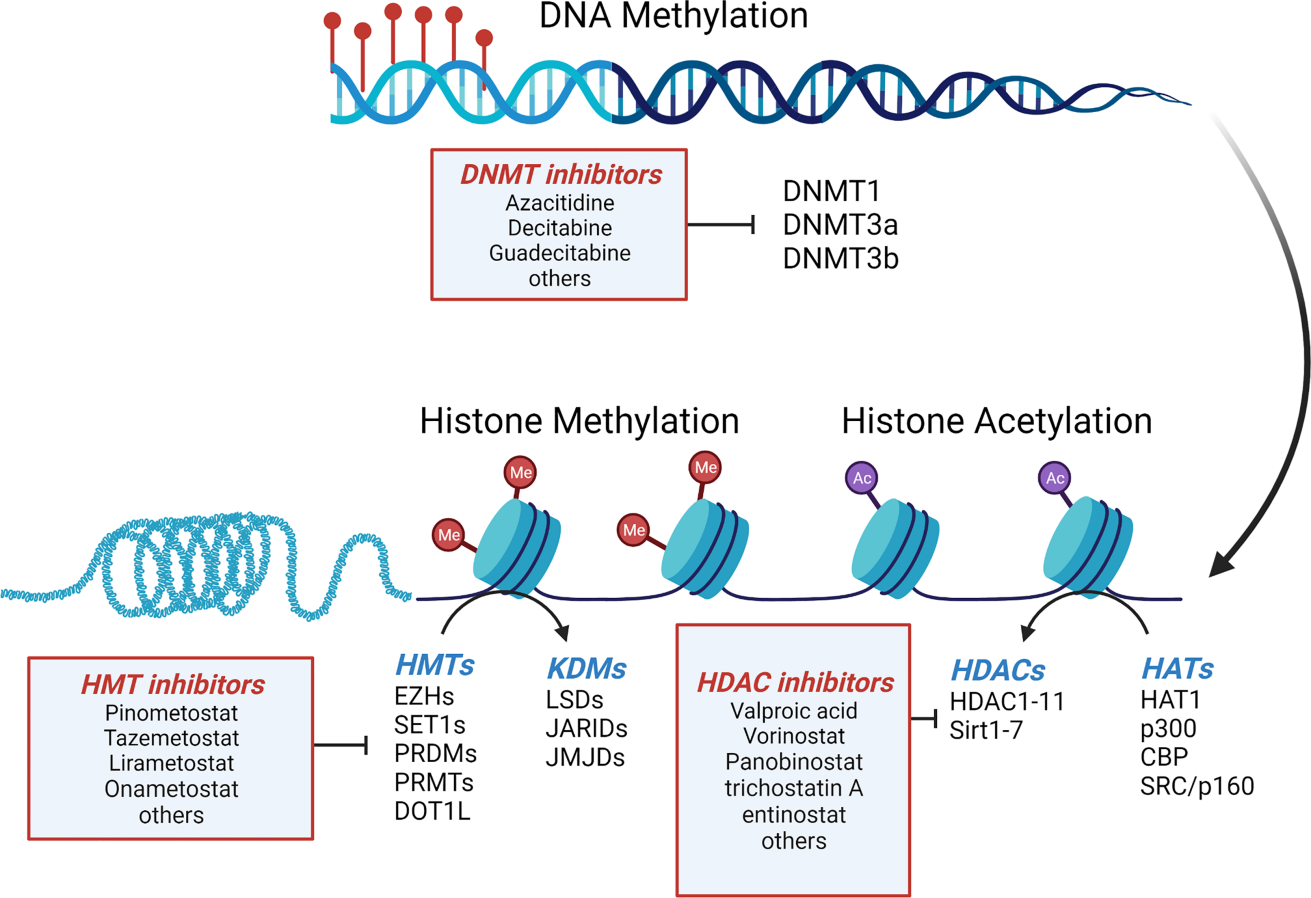
*Mechanisms of epigenetic regulation in GEP-NETs.* Regulation of gene expression in GEP-NETs is controlled in part by DNA methylation, histone methylation and histone acetylation. Addition of methyl groups to cytosines in CpG islands of genes, along with hypermethylation and hypoacetylation of histones results in gene silencing. DNMT, DNA methyltransferases; HATs, histone acetyltransferases; HDAC, histone deacetylases; KDM, histone lysine demethylases; HMT, histone methyltransferases. Adapted from “Epigenetic Levels” by BioRender.com (2022), Retrieved from https://app.biorender.com/biorender-templates.

DNA promoter methylation is one of the most well-studied epigenetic modifications, and the methylation of cytosine residues to 5-methylcytosine is a cornerstone of gene regulation and genomic homeostasis (**Figure 2**). DNA methylation alters the availability of transcription activation binding sites, inhibiting transcription. Moreover, additional inhibitory proteins, such as methyl CpG binding domain (MBD) and zinc finger proteins, bind to the methylated DNA further reinforcing transcriptional repression (44). There is also significant crosstalk with histone modifications (discussed below) where activating histone modifications prohibit binding of DNA methyltransferases (DNMTs) resulting in activation of transcription. The reverse is also true when repressive MBD proteins bound to methylated DNA prohibit histone modifications, resulting in transcriptional repression (44). In cancer, dysfunction of DNA methylation often results in genome-wide hypomethylation and increased chromosomal instability (CIN) alongside localized regions of DNA hypermethylation, specifically at CpG islands upstream of tumor suppressor genes which facilitate cancer growth and metastasis.

### 4.1 Role of DNMTs and Alterations in Global Methylation

In both healthy and cancer cells, DNA methylation is performed by the DNMT family of enzymes that transfer a methyl group from S-adenosyl-L-methionine to cytosine (45). Many cancers have overexpression or increased activity of DNMT1, DNMT3a and/or DNMT3b (46). One study examined the expression levels of DNMT1, DNMT3a and DNMT3b in a series of 63 GEP-NETs using immunohistochemistry (47). The authors found that DNMT1, DNMT3a and DNMT3b expression was detected in 87%, 81% and 75% of tumors, respectively. Further, the expression of DNMT3b was significantly elevated in poorly differentiated GEP-NECs when compared to well-differentiated GEP-NETs and NECs, and elevated in foregut tumors compared to mid- or hindgut NETs (47). The Simon et al. study performed DNA methylation profiling of the 57 pNENs (43 pNET, 14 pNEC) and was able to delineate pNET tumors from NEC based on t-distributed stochastic neighbor embedding (tSNE) analysis of methylation profiles (39).

Several early studies also examined global methylation patterns in small intestinal NETs (SI-NETs). A study of 20 primary SI-NETs and their metastases revealed decreased global methylation in metastases when compared to their primary tumors (48). Long interspersed nucleotide element 1 (LINE1) methylation, which can be used as a surrogate for global methylation patterns, was measured in 58 GEP-NETs (14 gastric, 15 pancreatic, 17 small intestinal, 8 appendiceal, and 4 colorectal). LINE1 hypomethylation was detected in 50% of gastric, 100% of pancreatic and colorectal, 82% small intestinal, and 87.5% of appendiceal tumors. Again, matched metastases exhibited decreased global methylation compared to their primary tumor counterparts (49). These smaller studies formed the foundation for a larger study performed by Karpathakis, et al. wherein the global landscape of 97 SI-NETs was analyzed using an integrated genomic approach. Based on this data, the authors categorized SI-NET tumors into three groups based on PFS after primary tumor resection. Twenty-one genes were identified with changes in methylation in as many as 85% of tumors (50). The more recent study by Yachida, et al. also evaluated methylation profiles and using unsupervised hierarchical cluster analysis of methylation status, divided their samples into three groups. pNETs were grouped into one group, non-pNETs in another, and the remaining group featured a CpG island methylator phenotype (CIMP) due to alterations in genes related to DNA methylation and/or due to MLH1 promoter hypermethylation (43).

### 4.2 Altered Promoter Methylation of Genes in GEP-NENs

Specific genes impacted by promoter methylation have been identified in GEP-NENs and include *RASSF1 (* 51–57), *CDKN2A (* 51, 52, 55, 58, 59), *TIMP3*, *MGMT (* 51, 52, 55, 60), *MLH1 (* 61), and *IGF2 (* 62) genes, among others. Methylation of these genes correlates with downregulated expression in GEP-NENs – often with molecular differences based on tumor tissue of origin. Downregulated expression of genes can also be the result of silencing by gene loss as noted by Capurso, et al. Many of the same genes silenced by hypermethylation, can also be genetically lost in GEP-NENs (63), suggesting a selective advantage for the tumor by inhibiting key proliferative processes within the cell.

#### 4.2.1 RASSF1

Ras-association domain gene family 1 (RASSF1) functions as a tumor suppressor gene that works through the cell cycle to arrest cells in G1 (64). Downregulation of RASSF1 is a result of epigenetic silencing by promoter methylation or allelic loss of 3p21.3, and results in cell cycle activation due to an accumulation of cyclinD1. The *RASSF1* gene includes a control region with two promoters, termed A and C, which control the production of eight RASSF1 isoforms. Selective methylation leads to only two of the isoforms being produced, RASSF1A and RASSF1C. RASSF1A not only regulates cellular proliferation, but also influences apoptosis and stabilization of microtubules, and downregulation has been reported in many cancers, including GEP NETs and other cancers of neuroendocrine origin (51–55, 64). Overexpression of RASSF1C occurs primarily in pNETs and is thought to inhibit beta-catenin within the Wnt signaling pathway, suggesting a role in development of pNETs (65).

In a collection of 48 pNETs, 87% of tumors were positive for aberrant methylation in at least one of 11 queried genes, with *RASSF1A* being hypermethylated in 75% of tumors when compared to non-tumor surrounding tissue. Furthermore, tumor size and aggressiveness correlated with methylation status, with tumors >5cm and those associated with lymph node or liver metastasis demonstrating a higher frequency of hypermethylation (55). Similarly, Malpeli, et al. noted an inverse correlation between RASSF1A expression and gene methylation when studying a group of 20 primary pNETs. In this study, the expression of RASSF1A was 6.8 times lower than normal tissue (57).

A study of 118 well differentiated fore- and midgut GEP-NETs (including 46 pancreatic, and 72 extrapancreatic) analyzed 11 genes and found that 71.3% of tumors had hypermethylation of *RASSF1A (* 51). Liu, et al. studied a different set of 47 GEP-NETs including pancreatic (n=16), nonilieal (including lung, gastric, duodenum, appendix, colon and rectum; n=15), and ilieal (n=16) tumors. Hypermethylation of *RASSF1A* was found in 57% of tumors with no significant correlation to site of origin, and was also associated with lymph node metastasis (52). Another study examined 62 gastrointestinal NETs and found aberrant methylation of *RASSF1A* in 32% of cases (all of foregut origin), cyclin D1 hyperexpression in 53% of cases, and deletion of 3p21.3 in 26% of tumors (53). Zhang, et al. analyzed 33 small bowel and matched metastatic tumors by methylation-specific PCR (56). *RASSF1A* was methylated in 60% of primary tumors and 84.8% of metastatic sites (56). Collectively, these studies suggest a significant role for methylation of *RASSF1A* in progression and metastasis of GEP-NENs. *In vitro* work from Pizzi, et al. shows that after treatment with 5-aza-2’-deoxycytidine or decitabine, RASSF1A mRNA was reexpressed (53), suggesting a treatment mechanism that may have promise for GEP-NENs.

#### 4.2.2 CDKN2A

The cyclin dependent kinase inhibitor 2A (*CDKN2A*) gene is located on chromosome 9p21.3 and generates several different transcripts through alternative splicing of the first exon. P16ink4a functions as an inhibitor of CDK4, and p14ARF functions as a stabilizer of p53 – both acting as tumor suppressors towards the common goal of cell cycle G1 checkpoint control. CDKN2A loss by promoter hypermethylation has been demonstrated in a number of cancers (66, 67), including GEP-NENs. A study from Liu, et al. indicated methylation present upstream of the *p14ARF* gene in 49% and upstream of *p16ink4a* gene 25% of the 47 well-differentiated NEN tumors analyzed (52). Bartsch, et al. identified only 2/17 (11.7%) pNET insulinomas with aberrant methylation of the *p16ink4a* promoter region (59). In a study of 48 well-differentiated pancreatic NETs, *p16ink4a* was hypermethylated in 40% of the cases, yet none of the pNETs in this study exhibited hypermethylation of *p14ARF (* 55). A contradicting study from 2004 indicates that in a study of 29 GEP-NENs, no hypermethylation of *p16ink4a* was observed (58) consistent with an additional study of 118 fore- and midgut NETs that found no evidence of *p16ink4a* methylation (51). In aggregate, these data suggest that hypermethylation of the *CDKN2A* promoter may contribute to tumorigenesis in only a subset of GEP-NENs.

#### 4.2.3 MGMT

O-6-methylguanine-DNA methyltransferase (MGMT) is a DNA repair protein that catalyzes the transfer of methyl groups from O-6-alkylguanine and to repair toxic lesions resulting from DNA alkylation. *MGMT* promoter methylation is linked to several cancer types, including GEP-NENs (68), and is predictive of tumor response to alkylating agents such as temozolomide. Indeed, *MGMT* was found to be hypermethylated in 40% of the tumors in a study of 48 pNETs (55). Another study of 118 well differentiated fore- and midgut GEP-NETs from 71 patients evaluated the promoter methylation of *MGMT*, and found that 16.1% of tumors had hypermethylation (51). Interestingly, within this group of tumors, gastrinomas had a significantly reduced *MGMT* promoter methylation when compared to insulinoma (p=0.023). *MGMT* methylation occurred in 13% of GEP-NENs (n=47), but not in those tumors of ileal neuroendocrine origin (52). Further, Kulke, et al. found MGMT deficiency in 19/37 (51%) pNETs by immunohistochemistry, while no MGMT deficiency was identified in GI-NETs. In this study, patients with pNETs had a much better response to temozolomide, and this association with clinical response may be due to MGMT deficiency (69). The ongoing MGMT-NET trial (NCT03217097) is studying whether MGMT methylation status is a predictive factor for response to alkylating agents (70).

#### 4.2.4 TIMP3

Other gene loci have been identified as having decreased gene expression as a result of promoter methylation in GEP-NENs. Wild, et al. identified methylation of tissue inhibitor of metalloproteinase-3 (*TIMP3*) in 8/18 pNET samples, along with corresponding loss or reduction in protein expression (71, 72). TIMP-3 could play a role in metastasis as evidenced by detection of methylation changes in 79% metastatic versus 14% of nonmetastatic pNETs. Similarly, in a study of 56 sporadic G1 and G2 pNETs, *TIMP3* methylation correlated with stage IV aggressive cancers with poor prognosis (73). Whereas TIMP3 expression has been noted in gastric cancer methylation of this gene in NENs of gastric/small intestinal origin has not been identified.

#### 4.2.5 Other Genes – IGF2, MLH1, APC

Insulin like growth factor 2 (*IGF2*) is an imprinted locus on chromosome 11p15.5 and overexpression has been linked to hypermethylation of the CpG islands in the differentially methylated region 2. Development of insulinoma has been linked to the loss of imprinting and overexpression of IGF2 (74, 75).

Other gene loci including *RAR*, *MLH1*, *E-cadherin*, *APC* and *p73* exhibited hypermethylated in 25%, 23%, 23%, 21% and 17% of tumors, respectively, in a study of 48 pNET (55). Mei, et al. observed *MLH1* methylation in 15/48 (31%) of insulinomas (76). Another study of 29 GEP-NENs found only one tumor hypermethylated at the *hMLH1* promoter but *APC* was hypermethylated in 65% (58).

### 4.3 MEN1 Status in DNA Methylation

In pNETs, the presence of mutations in *MEN1* also contributes to methylation status (77, 78). In a study of 29 pNETs, CpG hypermethylation correlated with MEN1 loss when compared to sporadic or VHL-associated pNETs (77). In a followup study, Tirosh, et al. examined 96 GEP-NEN samples by genome-wide methylation assays (querying a total of 835,424 CpGs). The methylome signature was compared between MEN1-related, VHL-related and sporadic pNET tumors. Unsupervised hierarchical clustering analysis identified two groups, with VHL-related tumors exhibiting marked DNA hypomethylation, and MEN1-related and sporadic tumors clustering together, although each group with a distinct methylation profile (79). Further, the authors identified elevated *APC* promoter hypermethylation in MEN1-related tumors (79) – a new finding that supported previous identification of APC mutations in 7/46 NET samples (80)

Coneman, et al. specifically analyzed MEN1-related pNETs for promoter methylation using methylation-specific multiplex ligation dependent probe amplification in 61 MEN1-related pNETs versus 34 sporadic pNETs (78). The cumulative methylation index (CMI; the sum of methylation percentages of all genes analyzed) showed no significant difference between the groups, but within the MEN1-related pNET group the CMI was significantly higher in larger pNETs. *CASP8* was one of only a few genes that demonstrated elevated promoter methylation in the MEN1-related pNET group compared to the sporadic pNETs (78).

Chan and colleagues found that 58% of pNETs (n=64), contained combined mutations in *ATRX*, *DAXX*, and *MEN1* (ADM mutant), a signature that also correlated with poorer clinical outcomes (81). Methylation analysis revealed a profile of hypermethylation in 59 genes in the ADM mutant tumors, with 13 of these genes demonstrating reduced expression. Seven genes were hypomethylated and overexpressed, including *APOH*, *CCL15*, *EMID2*, *PDZK1*, *HAO1*, *BAIAP2L2* and *NPC1L1 (* 81). Of note, the *PDX1* promoter in the ADM mutant tumors was hypermethylated at all 4 CpG islands and exhibited decreased expression compared to pNETs without the ADM mutations. The authors of the study suggest trans-differentiation of the tumor cells during development, a finding further supported by Cejas, et al. for nonfunctional pNETs (82).

### 4.4 DNA-Methylation Based Therapeutics

The cellular enzymes controlling the addition of DNA methylation marks are DNA methyl transferases (DNMTs) (83). The function, or more accurately the *dysfunction*, of these enzymes has an enormous impact on gene regulation in cancers, including GEP-NETs. To combat cancers driven by this mechanism, DNMT inhibitors have been developed, including azacitidine, decitabine, and guadecitabine (a dinucleotide prodrug of decitabine+guanine). All are approved by the FDA and function as nucleoside analogs to induce double stranded DNA breaks and subsequent apoptosis in actively dividing cells (84). These compounds are systemic in action and do not target a particular methylated CpG promoter region, but have been shown to promote re-expression of tumor suppressor genes. Efficacy of DNMT inhibitors *in vitro* shows promise: azacitidine caused a dose-dependent reduction in NET cell lines BON1, H727 and CNDT2.5 (85) and decitabine decreased proliferation of QGP1 cells (86). However, in other cancers the therapeutic potential of decitabine is decreased due to compound instability and dose-limiting hematological toxicities (87), suggesting the need for new compounds without these liabilities.

## 5 Chromatin Remodeling

DNA wraps around histone proteins to form nucleosomes which then compact to form chromatin. Histone proteins can be modified by the addition of acetyl, methyl, phosphoryl or ubiquitin groups to specific residues, often present in the N-terminal histone tails. Modification of histones in this way changes the ability of transcription factors to bind chromatin, altering gene expression. The histone complex normally includes two copies of H2A, H2B, H3 and H4 along with one copy of H1. Histone H1 is a family of linker histones that plays a role in chromatin stability, regulating gene expression, and participating in chromatin-based DNA repair. Histones can also have variants, resulting in modified histones, that have been identified in a variety of cancers, including GEP-NETs. A study of 13 primary NETs, showed the histone H1 family member H1x was overexpressed as measured by immunohistochemistry in tumor compared to surrounding normal tissue (88).

The novel identification of mutations in *ATRX* and *DAXX* in 17% and 25% of cases respectively, suggested alternative lengthening of telomeres (ALT) (89) as a contributing mechanism to pNET development. ATRX and DAXX are chromatin remodeling complex proteins that are required to incorporate histone variant H3.3 into chromosomal telomeres. In a study performed by Heaphy, et al, there was perfect correlation between the loss of ATRX/DAXX nuclear expression and the presence of ALT as measured by telomere-specific fluorescence *in situ* hybridization (89). Scarpa, et al. confirmed the mutation of *ATRX/DAXX* and the presence of ALT in 33/98 pNETs (38). Interestingly, the combination of mutations in *MEN1*, *ATRX* and *DAXX*, or *MEN1* with either *ATRX* or *DAXX* was associated with better outcomes compared to pNETs lacking these mutation combinations (37).

A study of structural rearrangements in pNETs identified chromosomal rearrangements in several genes involved in chromatin remodeling including *SETD2* (a histone lysine methyltransferase), *ARID2* (involved in chromatin structure modification), *KMT2C/MLL3* (histone lysine methyltransferase) and *SMARCA4* (part of the SWI/SNF chromatin remodeling complex) (38). *SETD2* and *ARID2* were identified as having mutations in 18 and 13% of advanced well differentiated pNETs, respectively (90).

### 5.1 Histone Acetylation

Histone remodeling by addition/removal of acetylation is facilitated by two enzymes (**Figure 2**). Histone deacetylases (HDACs) catalyze the removal of acetyl groups from specific lysine residues in histone proteins allowing for closing of chromatin and gene silencing. Histone acetyltransferases (HATs) do the opposite and mediate acetylation of histones resulting in a reduction in the positive charge on the surface of histones to loosen the attraction between DNA and the histones resulting in more open chromatin and gene accessibility for transcriptional activation (65).

Expression of all classes of HDACs (I, IIA, IIB, III and IV) was measured in a study of 57 pNETs on tissue microarrays (91). Significant upregulation (1.5 to >7 fold) of all HDACs was identified in pNET versus control, with the greatest increases in expression noted in the G3 tumors. The association of elevated HDAC expression with tumor grade, markers of proliferation and patient survival elevated inhibitors of HDACs as a potential therapy for pNETs and other neuroendocrine tumors (91). In addition to HDAC expression levels, functional histone acetylation has been studied in MEN1-related pNETs. After acetylation marks are written by HATs, these marks are read by the bromodomain and extraterminal (BET) protein. JQ1, a small molecule inhibitor of BET protein-protein interaction, decreased proliferation and apoptosis in an MEN1-dependent model of insulinoma (92).

The first histone deacetylase inhibitor (HDACi) to receive FDA approval was vorinostat in 2006 for the treatment of cutaneous T-cell lymphoma, and the history of HDACi development has been recently reviewed (93, 94). There is significant preclinical evidence in NET cell lines suggesting efficacy in GEP-NENs and *in vitro* studies performed with HDACi like trichostatin A, sodium butyrate, valproic acid and entinostat on pNET cell lines demonstrate dose dependent inhibition on cancer-related endpoints of cellular proliferation, apoptosis and cell cycle arrest (94–97). Preliminary clinical evidence evaluating panobinostat in GEP-NETs demonstrated stable disease in treated patients, but showed no effect on tumor volume (98). Additional clinical trials evaluating HDACi in GEP-NENs alone and in combination with mTOR inhibitors (everolimus/emsirolimus) or anti-PD-L1 immunotherapy (sintilimab) are in progress.

### 5.2 Histone Methylation

Methylation of core histones, particularly on H3 and H4, is a well-known phenomenon in cancer. Histone methylation can either be repressive or activating, depending on the histone, the location of the amino acid (lysine or arginine) to be modified, and the extent of methylation (i.e. di-, or trimethylation; me2 or me3, respectively). H3K9me2/3, H3K27me2/3 and H4K20me3 are known inhibitory methylation marks, while H3K4me2/3, H2K36me3 and K3K79me3 are known activating marks. Histone methylation is performed in the cell by histone methyl transferases (HMTs) and histone demethylases (HDMs), often in complexes with many cofactors (**Figure 2**).

An early study in NENs used immunohistochemistry to evaluate the presence of LSD1, a histone demethylating enzyme, and the methylating complex Ash2, using a tissue microarray that included 16 primary intestinal NECs. Strong immunostaining was present in 100% of NEC for Ash2 and 87% for LSD1. 93% of the tumors showed demethylation at H3K4, with only weak staining in surrounding normal tissue (99). Similarly, immunostaining for histone lysine methyltransferase enhancer of zeste homolog 2 (EZH2) indicated high differential expression of EZH2 in small intestinal NETs (100). EZH2 catalyzes H3K27me3 marks to function as a master regulator in a variety of cellular processes, including cancer, and its role in cancer is linked with high proliferation rates, metastasis and poor overall survival. *In vitro*, EZH2 inhibitors (CPI-1205/Lirametostat and GSK126) decreased cell viability, proliferation and migration capacity while increasing apoptosis in GI-NET cell lines (100). Histone methyltransferase inhibitors (HMTis) still in active development include EZH2 inhibitors CPI-1205/Lirametostat, Tazemetostat, and SHR2554, along with Pinometostat (DOT1L inhibitor), and should be considered the next frontier for pNET therapy (101).

Much of the work to understand histone methylation has been performed using MEN1/menin-related pNETs and model systems (102, 103). Menin plays a key role in epigenetic regulation by interacting with the histone lysine methyltransferases (MLL1/KMT2A and MLL2/KMT2B) (104) to influence writing of H3K4me3 methylation marks. Indeed, genome-wide studies of H3K4 methylation in pancreatic islets (105) indicate that loss of *Men1* alters the epigenetic landscape of its target genes such as insulin like growth factor binding protein 2 (*Igf2bp2*), *p18ink4c (CDKN2C)* and *p27kip1 (CDKN1B) (* 106). In mouse models, decreased expression of *Igfbp2* is accompanied by changes in H3K4 and H3K27 histone methylation at the promoter, changes that can be partially abrogated by deletion of the histone demethylase retinoblastoma binding protein 2 (*Rbp2*). This suggests that Rbp2 can reverse changes induced by the Menin/MLL complexes (105).

The histone lysine demethylase RBP2/KDM5A/JARID1A may also be involved in regulation of GEP-NEN tumorigenesis. RBP2 was overexpressed in a 20/25 human NEN primary tumors and metastases, with elevated expression most evident in the metastatic sites (107). Further *in vitro* study in NET cell lines demonstrated that overexpression of RBP2 significantly increased proliferation, migration, invasion and colony formation, whereas knockdown of RBP2 decreased the same parameters in a demethylase-dependent manner supporting the hypothesis that aberrant RBP2 expression and altered histone demethylation, is a frequent contributing factor to NET pathogenesis (108).

Histone modification does not always function independently and there is a unique interplay between DNMTs and histone methyltransferases in the context of malignancy (83). In general, active chromatin regions are characterized by acetylated histones and unmethylated DNA, while inactive regions of the genome are associated with methylated histones and methylated DNA (109). These two epigenetic mechanisms are intertwined such that DNA methylation guides histone modifications, and vice versa. Although not performed in GEP-NENs, two studies have demonstrated that EZH2-written H3K27 methylation marks directly control DNA methylation (110, 111), suggesting that abnormal methylation in tumor cells can be intimately linked with histone modification.

Protein arginine methyltransferase (PRMT5) functions with cofactors to mediate methylation of histones H2A and H4 at arginine 3, and H3 at arginine 8 (112). *In vitro* work in neuroendocrine cells demonstrates a direct binding between PRMT5 and menin at Gas1 and Gli1 promoters, resulting in repressive H4R3me2s methylation marks, decreased Gas1 and Gli1 expression, and decreased cell proliferation that is independent of classical Hedgehog signaling (30, 113). pNETs are known to have elevated GLI1 levels in the absence of menin (113), implying that inhibition of GLI1 could suppress formation of MEN1-related neuroendocrine tumors. Although not yet tested in GEP-NENs, loss of the enzyme methylthioadenosine phosphorylase (MTAP) due to genetic deletion of the nearby *CDKN2A* tumor suppressor may confer dependence on PRMT5 arginine methyltransferase activity as happens in other cancers (114). Inhibition of PRMT5 showed decreased cell viability in preclinical testing and as a result, several PRMT5 inhibitors (GSK3326595, AMG193, JNJ64619178/Onametostat, PF06939999, TNG908, PRT811, PRT543, and MRTX1719) have been developed and are in early phase clinical development for metastatic solid tumors and lymphoma demonstrating MTAP deletion. More work is needed in GEP-NENs to elucidate the role of PRMT5 and potentially other arginine methyltransferases in disease progression.

## 6 miRNA Regulation

miRNAs are small (18-23 nucleotide), noncoding RNA modulators of gene expression that are implicated in the control of critical processes involved in tumor development and metastasis by inducing inhibition and/or degradation of target mRNAs. miRNAs can be easily detected in biological fluids such as blood, saliva, urine, *et cetera* and also detected in exosomes (115). MiRNAs have potential utility as prognostic or diagnostic biomarkers as well as therapeutic targets, and recent data also suggests miRNAs may also play a role in therapy sensitivity and/or resistance (116).

Several miRNA profiling studies have been performed on NENs towards the goal of identifying miRNAs that hold promise as biomarkers to distinguish between the different tissues of origin within GEP-NENs as well as pancreatic acinar cell carcinoma (9, 117, 118). One case report analyzed the miRNA signature of normal pancreas, gastrinoma and pNET from a single MEN1 patient and highlighted sets of miRNAs that fall into two gene regulatory networks with distinct functional features (119). Klieser identified a “proliferative” miRNA signature that correlated with expression of HDAC as well as staging, grade and hormone activity in pNETs (120). Taken together, these data confirm much of the previous work focused on selected miRNAs such as miR-24 (121, 122), miR-21 (123), and others as reviewed recently (9) and is a critical step towards understanding the prognostic and diagnostic role of miRNAs.

Therapeutically, there are two current approaches in the preclinical stages of development that show promise for pNETs. The first uses stabilized miR-targeted locked nucleic acids that bind tightly to their target gene to block oncogenic miRNAs from interacting (124) and the second strategy uses antagomiRs, or miRNA mimetics (125) to restore function of tumor suppressing miRNAs. The field of nucleic acid-based therapeutics is growing exponentially based on past experiences in gene therapy, but challenges such as delivery modes and stability of RNAs remain. As biomarkers, miRNAs can be monitored as a measure of treatment response. For example, miRNA profiles correlated with pNET treatment response to metformin-induced decreased proliferation in cell lines (126).

## 7 Regulation of SSTR

SSTR2 and SSTR5 are the most frequently expressed SSTRs in GEP-NENs and are the target of somatostatin analog therapy. Recently, a natural antisense transcript, SSTR5-AS1, was identified that regulates the production and availability of SSTR5. Examination of expression levels in 15 pNET and adjacent normal tissue revealed that the expression level of SSTR5-AS1 was significantly higher in tumor samples, while SSTR5 expression was similar in both regions. Further studies in BON1 and QGP1 cells suggest that the expression may be linked as silencing SSTR5 with siRNA decreased expression of SSTR5-AS1. Methylation of CpG islands upstream of SSTR5 may be associated with upregulated expression of SSTR5 and SSTR5-AS1. SSTR5-AS1 promotes aggressiveness in pNET cells and may be involved in the limited response of these cells to pasireotide (127).

SSTR2 is also regulated by DNA methylation and histone modifications. BON1 and QGP1 cell lines both express SSTR2 at a relatively low level, despite mRNA levels greater than that seen in other cancer types and expression of SSTR2 is inversely related to the level of CpG island methylation (128). Further, treatment of BON1 cells with the DNMT inhibitor 5-aza-2’-deoxycytidine revealed stimulation of SSTR2 (129). Histone acetylation is also likely to be involved in regulation of SSTR2 levels in GEP-NETs and several *in vitro* studies in NET cell lines have investigated HDACi (romidepsin/FK228, vorinostat/SAHA, and AB3 along with valproic acid) with varying results (130, 131). Overall, these studies suggest the involvement of epigenetic mechanisms in the regulation of SSTR2 which may be capitalized upon to upregulate SSTR2 in patients with low SSTR levels prior to treatment with PRRT.

## 8 Discussion and Future Perspectives

GEP-NENs are a complex, very diverse family of tumors with many germline and somatic genetic abnormalities identified. In recent years, studies are revealing more about the epigenetic regulation that overlays the identified genetic mutations. Great strides have been made towards understanding the regulation of gene expression by DNA methylation, histone modifications and miRNAs in this group of tumors.

As with all new knowledge, our understanding of the epigenetics of GEP-NENs provokes addition questions about the etiology of these tumors, and the best approaches to take in patient management. There are newly revealed avenues of investigation that should be pursued to study the impact of therapies like PRMT5 inhibitors, EZH2 inhibitors, BET inhibitors like JQ1 (although chemical modification may be necessary to circumvent dose limiting adverse events), and combination strategies that could include HDAC inhibitors teamed with mTOR tyrosine kinase inhibitors, as has been done with other cancer types (132). More study is needed to understand the roles of different HDAC family members in NET development as proposed by Gagliano (94), as well as the efficacy and hopefully, reduced toxicity of selective HDAC inhibitors. For example, combination strategies such as EZH inhibitors teamed with metformin have been pursued *in vitro* in SI-NETs with growth arrest and increased apoptosis in GOT1 spheroid models (100). EZH2 inhibitors combined with dopamine receptor D2 antagonists showed measurable promise in an organoid model of androgen receptor-insensitive neuroendocrine prostate tumors (133). Finally, mTOR inhibitors combined with azacitidine also showed antiproliferative and apoptotic activity in medullary thyroid carcinoma (134), with the suggestion that this combination may also have efficacy in neuroendocrine tumors for which mTOR inhibitors are approved.

The concept of stochastic epigenetic mutations, or a measure of life-course accumulation of exposure-related, epigenetic damage as a function of evolution, is also understudied in cancers in general. Feinberg and Irizarry originally proposed this model wherein epigenetic variation as a response to environmental stimuli is a mechanism for inherited, evolutionary adaptation (135). Recent studies have demonstrated correlations between SEM and a higher risk of breast cancer, liver cancer, lung cancer, and mature B-cell neoplasms (136, 137). This has not been studied in GEP-NENs.

Further, there are other completely unexplored territories including the interplay of signaling pathways. For example, the role of genes like *RASSF1A* that are known to have decreased expression in NETs due to promoter hypermethylation or gene loss, and how these proteins interact with signaling pathways such as Notch. A recent publication demonstrates how loss of RASSF1A expression allows for tumor dedifferentiation and proliferation as a result of accumulated Hes1, suggesting a role for RASSF1A/Notch crosstalk in GEP-NENs that has not yet been explored (138, 139). Further, the lysine demethylase protein KDM5A/RBP2, shown to be overexpressed in GEP-NENs (107), is a key component of the CSL repressor complex in Notch signaling and also may play a role in epigenetic regulation of cancer cell proliferation and stemness. All of this evidence provides a foothold for epigenetic changes as a key player in GEP-NEN development, progression, metastasis, and response to treatment. The large scale omics approaches are beginning to reveal the mechanisms, both genetic and epigenetic, of tumorigenesis. Such comprehensive, integrated approaches teamed with appropriately powered clinical trials based on specific, molecular-based therapeutics is expected to have a direct clinical impact on the management of GEP-NENs.

## Author Contributions

The author confirms being the sole contributor of this work and has approved it for publication.

## Funding

This work is supported by the Louisiana State University Health Sciences Center School of Medicine, Department of Genetics.

## Conflict of Interest

The author is a member of the Amgen Neuroendocrine Tumor Expert Advisory Board and receives an honorarium for this service.

## Publisher’s Note

All claims expressed in this article are solely those of the authors and do not necessarily represent those of their affiliated organizations, or those of the publisher, the editors and the reviewers. Any product that may be evaluated in this article, or claim that may be made by its manufacturer, is not guaranteed or endorsed by the publisher.
